# Supplementary material for: Principal Component Analysis applied directly to Sequence Matrix
Source: Sci Rep. 2019 Dec 17;9:19297. doi: 10.1038/s41598-019-55253-0 (PMC6917774; doi:10.1038/s41598-019-55253-0)
Supplement: Supplementary file 1 — dataset 1 [file 41598_2019_55253_MOESM1_ESM.zip › 55253/41598_2019_55253_MOESM1_ESM_revised.zip!/41598_2019_55253_MOESM1_ESM/supplement/S1/index.html]

S1


## Figure S1

Click images to enlarge

| A | B |
| --- | --- |
|  |  |

  

**Fig. S1. Normal QQ plot of PC scores.**   
Those were made to help choosing the threshold (dotted lines) for to define specific bases.   
**A**: PC1, **B**: PC2 of lion data (Fig. 3A).
