# Supplementary material for: Principal Component Analysis applied directly to Sequence Matrix
Source: Sci Rep. 2019 Dec 17;9:19297. doi: 10.1038/s41598-019-55253-0 (PMC6917774; doi:10.1038/s41598-019-55253-0)
Supplement: Supplementary file 1 — dataset 1 [file 41598_2019_55253_MOESM1_ESM.zip › 55253/41598_2019_55253_MOESM1_ESM_revised.zip!/41598_2019_55253_MOESM1_ESM/supplement/S2/FigS2_BC.html]

 
RGL model


You must enable Javascript to view this page properly.

  
Drag mouse to rotate model. Use mouse wheel or middle button
to zoom it.

---

  
Object written from rgl 0.99.16 by writeWebGL.
