# Supplementary material for: Principal Component Analysis applied directly to Sequence Matrix
Source: Sci Rep. 2019 Dec 17;9:19297. doi: 10.1038/s41598-019-55253-0 (PMC6917774; doi:10.1038/s41598-019-55253-0)
Supplement: Supplementary file 1 — dataset 1 [file 41598_2019_55253_MOESM1_ESM.zip › 55253/41598_2019_55253_MOESM1_ESM_revised.zip!/41598_2019_55253_MOESM1_ESM/supplement/S3/index.html]

S3


## Figure S3

Click images to enlarge

| A | B |
| --- | --- |
|  |  |
| C | D |
|  |  |
| E | F |
|  |  |

  

**Fig. S3. Effect of alternative alignment methods to the bacteria samples.**  
The methods were performed with default parameters. **A-D**: direct PCA, **E** and **F**: NJ method.   
**A**: ClustalW, **B**: MAFFT, **C** and **E**: DECHIPHER, **D** and
**F**: DECHIPHER with gap penalty 1/3 of the default.

## Contributions

| G | H |
| --- | --- |
|  |  |

  

MUSCLE, default. **G**: contribution, **H**: tangent regression.
