# Supplementary material for: Principal Component Analysis applied directly to Sequence Matrix
Source: Sci Rep. 2019 Dec 17;9:19297. doi: 10.1038/s41598-019-55253-0 (PMC6917774; doi:10.1038/s41598-019-55253-0)
Supplement: Supplementary file 1 — dataset 1 [file 41598_2019_55253_MOESM1_ESM.zip › 55253/41598_2019_55253_MOESM1_ESM_revised.zip!/41598_2019_55253_MOESM1_ESM/supplement/S6/index.html]

S6


## Figure S6

Click images to enlarge

| A | B |
| --- | --- |
|  |  |
| C | D |
|  |  |
| E | F |
|  |  |

  

**Fig. S6. Effect of different gap penalties in direct PCA of human samples.**  
White circles: default parameter; Black circles: 1/3 of the default parameter. **A-F**: PC1 and PC2 to PC11 and PC12, respectively.
