# Supplementary material for: Principal Component Analysis applied directly to Sequence Matrix
Source: Sci Rep. 2019 Dec 17;9:19297. doi: 10.1038/s41598-019-55253-0 (PMC6917774; doi:10.1038/s41598-019-55253-0)
Supplement: Supplementary file 1 — dataset 1 [file 41598_2019_55253_MOESM1_ESM.zip › 55253/41598_2019_55253_MOESM1_ESM_revised.zip!/41598_2019_55253_MOESM1_ESM/supplement/S7/index.html]

S7


## Figure S7

Click images to enlarge

| A | B |
| --- | --- |
|  |  |

  

**Fig. S7. Adjustment of distances for human samples.**  
PC1 and PC2 for adjusted distances (**A**). Histogram of compensation ratio (**B**).
