# Supplementary material for: Principal Component Analysis applied directly to Sequence Matrix
Source: Sci Rep. 2019 Dec 17;9:19297. doi: 10.1038/s41598-019-55253-0 (PMC6917774; doi:10.1038/s41598-019-55253-0)
Supplement: Supplementary file 1 — dataset 1 [file 41598_2019_55253_MOESM1_ESM.zip › 55253/41598_2019_55253_MOESM1_ESM_revised.zip!/41598_2019_55253_MOESM1_ESM/supplement/S8/index.html]

S8


## Figure S8

Click the image to enlarge

**Fig. S8.**  
PCA can be recognized as a rotation of a data matrix.   
Information, including the relationships among samples, are maintained.   
  
The direction of the rotation is determined by SVD, to fit the diagonal axes of PC.   
  
Here, the relationship between a pair of samples (vertical solid line) was tilted by the rotation.   
The line is projected to PC1 and PC2; in this case, PC2 contains larger projections.   
The square root of the sum of the squares of the projections is the original length (distance) of the line.   
  
The distance shows the rate of difference at each site (base or residue) on average;   
if it is 0.1, then 1 out of 10 sites are different.
