# Supplementary material for: Principal Component Analysis applied directly to Sequence Matrix
Source: Sci Rep. 2019 Dec 17;9:19297. doi: 10.1038/s41598-019-55253-0 (PMC6917774; doi:10.1038/s41598-019-55253-0)
Supplement: Supplementary file 1 — dataset 1 [file 41598_2019_55253_MOESM1_ESM.zip › 55253/41598_2019_55253_MOESM1_ESM_revised.zip!/41598_2019_55253_MOESM1_ESM/supplement/S9/index.html]

S9


## Figure S9

Click the image to enlarge

### Simulation: property of samples and PCA

#### Random walk

|  |  |  |  |
| --- | --- | --- | --- |
|  |  |  |  |
| PC1 | PC2 | PC3 | PC4 |
|  |  |  |  |
|  |  |  |  |
| contribution | tangent | *ƒÆ* and PC |  |
|  |  |  |  |

The random walk samples, which mimic a sequentially altered evolution, give Fourier series in each PC with constant oscillations. The contributions attenuate quickly and correlate with tan *ƒÆ* (1/2 π > *ƒÆ* ≥ 0).

#### Randomly altered

|  |  |  |  |
| --- | --- | --- | --- |
|  |  |  |  |
| PC1 | PC2 | PC3 | PC4 |
|  |  |  |  |
|  |  |  |  |
| contribution | tangent |  |  |
|  |  |  |  |

Randomly altered samples, which mimic a diverged group, would give a randomly distributed PC for samples. The contributions attenuate evenly and the tangent regression bends.

#### R code
