# Supplementary material for: Principal Component Analysis applied directly to Sequence Matrix
Source: Sci Rep. 2019 Dec 17;9:19297. doi: 10.1038/s41598-019-55253-0 (PMC6917774; doi:10.1038/s41598-019-55253-0)
Supplement: Supplementary file 1 — dataset 1 [file 41598_2019_55253_MOESM1_ESM.zip › 55253/41598_2019_55253_MOESM1_ESM_revised.zip!/41598_2019_55253_MOESM1_ESM/supplement/supplement.html]

Supple


## Supplementary Information

for **Principal Component Analysis applied directly to Sequence Matrix**  
Tomokazu Konishi, Shiori Matsukuma, Hayami Fuji, Daiki Nakamura, Nozomi Satou, and Kunihiko Okano

## Figures

|  |  |
| --- | --- |
| S1 | Normal QQ plot of PC scores. |
| S2 | Neighbor joining tree of lions and human. |
| S3 | Effect of alternative alignment methods to the bacteria samples. |
| S4 | Effect of alternative alignment methods to the lion samples. |
| S5 | Effect of alternative alignment methods to the human samples. |
| S6 | Effect of different gap penalties in direct PCA of human samples. |
| S7 | Adjustment of distances for human samples. |
| S8 | PCA and the scores. |
| S9 | Simulation: property of samples and PCA. |

  

## Tables

|  |  |
| --- | --- |
| S1 | sPC for bases for bacteria samples. |
| S2 | sPC for bases for human samples. |
| S3 | Human sequences. |
| S4 | Lion sequences. |
| S5 | Bacteria sequences. |
| S6 | Alinged lion sequences. |

  

## R codes

|  |  |
| --- | --- |
| codes | The R codes in ascii text. |
| Trial Kit (zipped) | A starters kit. |
